# Neuroprotective Role of Omega-3 Fatty Acids: Fighting Alzheimer’s Disease

**DOI:** 10.3390/molecules30153057

**Published:** 2025-07-22

**Authors:** Mervin Chávez-Castillo, María Paula Gotera, Pablo Duran, María P. Díaz, Manuel Nava, Clímaco Cano, Edgar Díaz-Camargo, Gabriel Cano, Raquel Cano, Diego Rivera-Porras, Valmore Bermúdez

**Affiliations:** 1 Endocrine and Metabolic Diseases Research Center, School of Medicine, University of Zulia, Maracaibo 4001, Venezuela; mervinch12@gmail.com (M.C.-C.); paulagy26@gmail.com (M.P.G.); pabloduran1998@gmail.com (P.D.); mariadiazalbornoz@hotmail.com (M.P.D.); ma-nuelnava_14@hotmail.com (M.N.); antioxidante48@gmail.com (C.C.); 2 Facultad de Ciencias Jurídicas y Sociales, Universidad Simón Bolívar, Cúcuta 540006, Colombia; edgara.diaz@unisimon.edu.co; 3 Freie Universität Berlin, Institut für Pharmazie, Königin-Luise-Strasse 2-4, 14195 Berlin, Germany; gabriel.simon.cano@fu-berlin.de; 4 Clínica General del Norte, Grupo de Estudio e Investigación en Salud, Barranquilla 080001, Colombia; raquelamiracano@gmail.com; 5 Universidad de la Costa, Departamento de Productividad e Innovación, Barranquilla 080001, Atlántico, Colombia; drivera23@cuc.edu.co; 6 Universidad Simón Bolívar, Facultad de Ciencias de la Salud, Centro de Investigaciones en Ciencias de la Vida, Barranquilla 080001, Atlántico, Colombia

**Keywords:** polyunsaturated fatty acids, Alzheimer’s disease, neuroprotective molecules, anti-inflammatory activity

## Abstract

Alzheimer’s disease (AD) is one of the main causes of dementia, with an exponential increment in its incidence as years go by. However, since pathophysiological mechanisms are complex and multifactorial, therapeutic strategies remain inconclusive and only provide symptomatic relief to patients. In order to solve this problem, new strategies have been investigated over recent years for AD treatment. This field has been reborn due to epidemiological and preclinical findings that demonstrate the fact that omega-3 polyunsaturated fatty acids (ω-3 PUFAs) can be promising therapeutic agents because of their anti-inflammatory, antioxidant, and neurogenic-promoting activities, thus allowing us to classify these molecules as neuroprotectors. Similarly, ω-3 PUFAs perform important actions in the formation of characteristic AD lesions, amyloid-β plaques (Aβ) and neurofibrillary tangles, reducing the development of these structures. Altogether, the aforementioned actions hinder cognitive decline and possibly reduce AD development. In addition, ω-3 PUFAs modulate the inflammatory response by inhibiting the production of pro-inflammatory molecules and promoting the synthesis of specialised pro-resolving mediators. Consequently, the present review assesses the mechanisms by which ω-3 PUFAs can act as therapeutic molecules and the effectiveness of their use in patients. Clinical evidence so far has shown promising results on ω-3 PUFA effects, both in animal and epidemiological studies, but remains contradictory in clinical trials. More research on these molecules and their neuroprotective effects in AD is needed, as well as the establishment of future guidelines to obtain more reproducible results on this matter.

## 1. Introduction

Alzheimer’s disease is the most frequent neurodegenerative disorder worldwide and the most prevalent cause of neurocognitive impairment in the elderly population [1], affecting a total of 24 million people, with an estimated duplication of these numbers by 2040 [2]. This clinical entity has a multifactorial origin, conditioned by genetic and environmental factors, in addition to the age of the individual [3]. The disease is characterised by progressive loss of neurocognitive functions, mainly memory, eventually progressing to impairment in other spheres, such as voluntary motricity and vegetative functions [4].

In general, the typical structural changes in AD are a product of the abnormal accumulation of misfolded beta amyloid and tau proteins [2], which leads to the formation of lesions known as amyloid plaques and neurofibrillary tangles, generally located within the temporal lobe. These lesions disrupt neuronal synapses and promote the beginning of inflammatory processes that contribute to disease progression [5]. This sequence of events is known as the amyloid cascade hypothesis (ACH). Despite its wide acceptance, the exact alterations and variability in disease progression remain to be completely elucidated [2]. Thus, effective therapeutic strategies have been difficult to achieve.

Accordingly, the results obtained with current treatments based on the ACH do not seem to disrupt or avoid progressive neurodegeneration. In the hope of sorting out this issue, new approaches focused on inflammatory mechanisms involved in AD have been established, along with AD prevention through interventions on modifiable risk factors such as diet and lifestyle [6]. In this sense, ω-3 PUFAs, key components of a healthy diet, have emerged as possible therapeutic agents for AD treatment. These molecules are essential components of neurons’ lipidic membrane and participate in the modulation of multiple functions, such as cell migration regulation and neurotransmission, as well as inflammatory markers and neuroprotection against oxidative stress. The previously mentioned functions have been confirmed by clinical and epidemiological trials, which have identified an association between ω-3 polyunsaturated fatty acid (ω-3 PUFAs)-rich diets and a lower risk of the development and progression of cognitive impairment [7,8].

Hence, this review aims to explore current knowledge on the neuroprotective mechanisms of ω-3 PUFAs and their implications on AD development, as well as demonstrating the effectiveness of ω-3 PUFA supplementation in patients with Alzheimer’s disease.

## 2. Materials and Methods

This review provides compiled information regarding the function of ω-3 PUFAs in AD through an extensive and non-systematic literature search in the Scopus, EMBASE, PubMed, ISI Web of Science, ScienceDirect, Medline, Cochrane Library Plus, and Google Scholar databases, from inception to July 2025. Only articles in Spanish and English were used. No restrictions were made based on the type of study. Scientific articles from high-impact journals were selected: Q1, Q2, and Q3. The studies used for the preclinical and clinical evidence section were selected, predominantly, based on the clarity and reproducibility of their methodology and scientific quality. The search strategy included terms such as “Alzheimer’s disease”, “PUFAs”, “neuroprotection”, and “inflammation” combined with Boolean operators (AND/OR).

## 3. Results

### 3.1. Key Neurodegenerative Mechanisms in Alzheimer’s Disease

**Figure 1:**
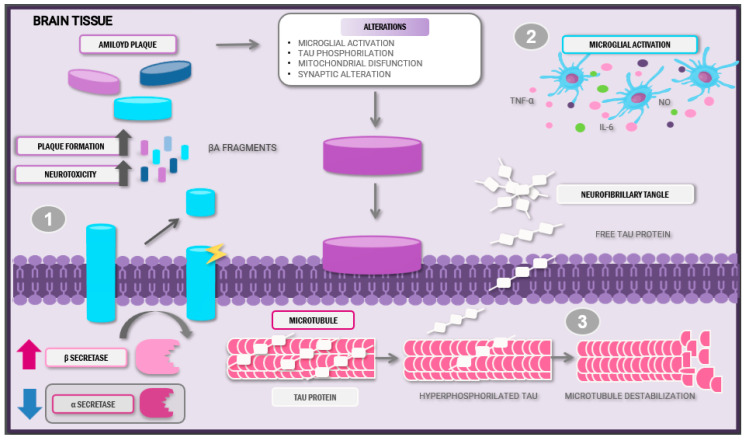
Anatomopathological alterations and pathophysiology of Alzheimer’s disease. Abbreviations: Aβ: amyloid beta; IL-6: interleukin-6; TNF-α: tumour necrosis factor; NO: nitric oxide.

Alzheimer’s disease evolution begins many years before the initiation of clinical symptoms [9]. Within the different theories concerning AD development, the ACH explains that the formation of senile plaques, composed of misfolded Aβ proteins of 40–12 amino acids, as a product of altered amyloid precursor protein (APP) cleavage by β-secretase [10]. These lesions initially develop in the basal, temporal, and orbitofrontal regions of the neocortex. As the disease progresses, the lesions expand to the hippocampus, amygdala, diencephalon, and basal ganglia [11]. The deposition of plaques induces mitochondrial damage, homeostatic dysregulation, and synaptic dysfunction, along with the hyperphosphorylation of tau proteins. As a result, the proteins lose affinity for microtubules and instead form conglomerates of misfolded tau proteins, leading to the formation of neurofibrillary tangles. These structures aggravate axonal transport and mitochondrial dysfunction, with the subsequent loss of synaptic function [12] (Figure 1).

Amyloid plaque and neurofibrillary tangle formation characterises Alzheimer’s disease. (1) These plaques are a product of the altered cleavage of precursor amyloid protein by the β-secretase enzyme, thus provoking Aβ neurotoxic peptide release and posterior aggregation. (2) Abnormal Aβ accumulation induces microglial activation and pro-inflammatory cytokine release. (3) The hyperphosphorylation of tau proteins leads to microtubule destabilisation and loss of synaptic function. This sequence of events creates a pro-inflammatory and neurotoxic state, aggravating disease evolution.

Due to the high toxicity and capacity to induce neuronal damage, Aβ and hyperphosphorylated tau protein can lead to microglial cell infiltration around amyloid plaques [11]. Once activated, these cells can adopt different phenotypes: M1, induced by interferon-gamma (IFN-γ) and lipopolysaccharide (LPS) stimulation, producing pro-inflammatory cytokines release, and the M2 phenotype, activated by interleukins (ILs) and characterised by the expression of anti-inflammatory molecules such as transforming growth factor β (TGF-β) and extracellular matrix molecules [13].

During the early phases of the disease, the lesions induce microglial polarisation into the M1 phenotype via specific receptors, such as CD14, CD36, and Toll-like receptors (TLRs), which facilitates the release of pro-inflammatory cytokines. These molecules recruit other cells that proceed to organise themselves to phagocytose the pathogen [14]. However, the continual production of these aberrant proteins and their interactions with the microglia do not allow a resolution phase to occur; instead, they promote exacerbated inflammatory responses and defective Aβ plaque removal [15].

Moreover, abnormal Aβ accumulation induces impaired mitochondrial membrane potential by disrupting its homeostasis and enzymatic activity. Aβ enters the mitochondria via the translocase of the outer membrane complex, leading to excessive production of reactive oxygen species and limiting the antioxidant system’s ability to neutralise them, consequently resulting in oxidative stress, subsequent neuronal damage, and further mitochondrial dysfunction [16].

Additionally, epigenetics plays a significant role in Alzheimer’s disease progression. Epigenetic modifications (e.g., DNA methylation and histone alterations) can be influenced by both modifiable (e.g., diet and environmental toxins) and non-modifiable factors (e.g., ageing). Among the non-modifiable genetic factors, mutations in the presenilin genes (PSEN1 and PSEN2), which encode essential subunits of the γ-secretase complex, promote the production of neurotoxic amyloid-β (Aβ42) by altering the proteolytic processing of amyloid precursor protein (APP). Furthermore, the APOE ε4 allele, which impairs Aβ clearance and accelerates its aggregation, is a major genetic risk factor for amyloid plaque deposition [17,18]. Within the non-modifiable factors, APP genetic mutations, presenilin 1 (PSEN1), and presenilin 2 (PSEN2) favour the production of toxic Aβ forms. Moreover, the presence of allele 4 of the APOE protein, which binds to Aβ, and its subsequent polymerisation are also considered relevant factors in plaque deposition [18]. Furthermore, age is considered the main factor in AD development, since this phenomenon is linked to diminished DNA methylation capacity in the brain, leading to mitochondrial decline. In consequence, mitochondrial dysfunction affects the expression and processing of APP, triggering Aβ accumulation [19,20]. On the other hand, acquired factors such as strokes and dyslipidaemias are linked to oligemia, brain–blood barrier dysfunction, and the accumulation of neurotoxic molecules that produce neuronal damage [21].

Lastly, modifiable variables, such as diet and exercise, are associated with beneficial and neuroprotective effects against AD development. Regarding physical activity, its neuroprotective effects could be produced by the release of neurotrophic factors such as insulin-like growth factor (IGF-1), nerve growth factor (NGF), and vascular endothelial growth factor (VEGF), since they exhibit antioxidant properties and promote cerebral perfusion [22]. Likewise, diets that include abundant unsaturated fats and antioxidants contain essential neuroprotective components, such as vitamins and PUFAs. These molecules can stabilise the neuronal membrane since they promote Aβ peptide elimination and phospholipid synthesis to improve and preserve synaptic functions [23,24].

### 3.2. Omega-3 Fatty Acids as Neuroprotective Agents

The nervous system is rich in fatty acids, and PUFAs, particularly, constitute about 35% of this tissue [25]. Thus, incorporating such molecules is necessary to maintain nervous cells adequately. Among fatty acids, the ω-3 series is mainly represented by eicosapentaenoic acid (EPA) and docosahexaenoic acid (DHA) [26]. These acids are found in seafood and vegetable sources (ALA) [27]. Both DHA and EPA are part of the lipid membrane and act as substrates for molecule synthesis, improving cognitive processes and performing neuroprotective activities, due to their anti-inflammatory, antioxidant, and neuromodulator activities [28].

ω-3 PUFAs, and particularly DHA, display multiple actions that counteract amyloid plaque formation. Among the mechanisms involved, their capacity to affect amyloidogenic and non-amyloidogenic processing is highly relevant. Regarding the first pathway, PUFAs’ main actions consist of 1. γ-secretase suppression, via PESEN1; 2. β-secretase inhibition; 3. BACE1 altered signalling; and 4. BACE1 downregulation through PPAR activation. Meanwhile, non-amyloidogenic processing is in charge of 5. α-secretase stabilisation, 6. ADAM17 increase, and 7. the interstitial removal of Aβ peptides via aquaporins.

#### 3.2.1. Anti-Inflammatory Role

In pathological conditions such as AD or ageing, pro-inflammatory states as a result of microglial dysfunction are a signature feature [29]. Inflammation is a defence mechanism designed to repair and protect tissues [30] which is usually controlled and self-limited. Nevertheless, under certain circumstances, it can turn into a chronic process and contribute to tissue damage [31].

Different hypotheses have been proposed to explain the anti-inflammatory mechanisms of ω-3 PUFAs [32]. Among these, microglial modulation is highlighted, since microglia are capable of inducing M2 phenotype polarisation and thus decrease the production of pro-inflammatory cytokines such as tumour necrosis factor alpha (TNF-α), interleukin-1beta (IL-1β), and interleukin-6 (I-6) [33]. PUFAs presumably diminish factor nuclear kappa B subunit (IκB) phosphorylation and induce peroxisome proliferator-activated receptor (PPARγ), consequently inhibiting NF-κB translocation to the cellular nucleus [34]. Simultaneously, PUFAs promote the disruption of the P38 of mitogen-activated protein kinase (MAPK)7 signalling pathway [35], involved in the biosynthesis of pro-inflammatory cytokines like TNF-α and IL-1β [36].

Moreover, cyclooxygenase (COX) and lipoxygenase (LOX) enzymes catalyse the oxidation of EPA and DHA molecules, resulting in compounds specialised in inflammation resolution, known as specialised pro-resolving lipid mediators (SPMs) [31]. Within this group, we have molecules such as E series resolvins (RvE1-3), derived from EPA; D series resolvins (RvD1-6); maresins (Mar1-2); and neuroprotectins (NPD1), derived from DHA [37].

Particularly, resolvins (Rvs) are bioactive compounds with chemoselective and focalised activity, whose pro-resolutive and anti-inflammatory effects are exerted through G protein-coupled receptors (GPCRs) [38]. Molecules derived from the E series (RevE1-E3) act as ChemR23 agonists. This receptor is located in microglia and neurons widely distributed within the prefrontal cortex and hippocampus, where it increases the phagocytosis of apoptotic cells, diminishes pro-inflammatory cytokines, and induces beneficial resolving signals [39]. In addition, resolvins act as BLT-1 agonists in order to block polymorphonuclear (PMN) cell chemotaxis and NF-kB activation [40]. RvD exerts similar effects regarding PMN cell infiltration regulation [41], NF-κB activation, and the negative modulation of pro-inflammatory cytokine production via microRNA induction [31].

Concurrently, NPD1 modulates anti-apoptotic protein activity and decreases caspase-3 activation induced by oxidative stress, thus promoting cellular survival. Furthermore, this molecule inhibits COX-2 and NF-κB expression in order to regulate pro-inflammatory molecules [42]. The neuroprotective effects associated with Mars molecules range from the downregulation of inflammatory markers through NF-κB pathway inhibition [43] to the decrease in the levels of neuronal death promoter CD40 and the increase in tissue regeneration [44].

#### 3.2.2. Role of ω-3 PUFAs in Oxidative Stress

The accumulation of the Aβ peptide and hyperphosphorylated tau proteins, along with the persistent activation of microglial cells observed in AD patients, creates an environment of abnormally high oxidative stress levels in the nervous system [28]. Therefore, multiple mechanisms by which ω-3 PUFAs might counteract this environment have been proposed. Their antioxidant activities are a product of cell migration and the induction of nuclear factor erythroid 2-related factor 2 (Nrf-2). PUFAs bind to the antioxidant response element (ARE) and augment the gene transcription of antioxidant enzyme heme oxygenase (HO-1), nicotinamide adenine dinucleotide (NAD), and quinone oxidoreductase 1 (Nqo1) [45]. PUFAs are also able to induce microglial polarisation into the M2 phenotype, favouring phagocytic activity and simultaneously hindering the production of oxidative stress-promoting factors, such as nitric oxide (NO) and reactive oxygen species (ROS) [25].

Similarly, SPMs also act as oxidative stress antagonists through ROS reduction, since these molecules activate antioxidant enzymes that promote homeostatic balance. Rvs suppress NADPH oxidase NOX2 in microglia and antagonise BLT1, thereby regulating ROS production and cell apoptosis [46]. Similarly, NDP1 induces the phosphoinositide 3-kinase/Akt pathway, enabling the activation of cell survival mechanisms [47]. The antioxidant effects linked to Mars result from the modulation of the Nrf-2/HO-1 pathway [48].

#### 3.2.3. Role of PUFAs in Neuronal Generation

After embryogenesis and early postnatal life, neurogenesis is a process exclusively maintained in two areas of the adult brain [49]: the subventricular zone of the lateral ventricles and the subventricular layer of the hippocampus dentate gyrus [50]. However, ageing, pro-inflammatory chronic states, and oxidative stress can potentially diminish this process [51].

In vivo and in vitro studies have demonstrated that DHA supplementation induces an increase in the axonal growth marker Growth-Associated Protein 43 (GAP-43), thereby promoting the growth and ramification of hippocampal dendrites [52]. Furthermore, DHA induces an increase in synapsin levels [53] and binds to the GPCR40 receptor, leading to a cascade of events that culminate in calcium mobilisation, the activation of protein kinase C (PKC), and increased production of substrates required for synaptogenesis.

Moreover, both EPA and DHA exert modulating effects on neurogenesis via transcription factors Hes-1 and Hes-6, which act through a positive feedback cycle to promote neuronal differentiation [50]. Multiple DHA neurogenic effects can be explained by their active mediators, specifically docosahexaenoyl ethanolamandine (DHEA). This endocannabinoid metabolite activates protein kinase A (PKA), allowing the subsequent differentiation of neuronal stem cells [54].

### 3.3. Preclinical Evidence of Omega-3 Fatty Acids in Alzheimer’s Disease

It is important to note that the neuroprotective effect of ω-3 PUFAs in AD and other neurodegenerative diseases is primarily attributed to their anti-inflammatory, antioxidant, and neuronal growth-promoting mechanisms. In addition to this, DHA (one of the derivatives of PUFAs) has been shown to exhibit molecular mechanisms capable of acting on two key processes of AD development: the formation of Aβ plaques and the aggregation of tau protein. However, further preclinical research is required to demonstrate the role of EPA, another PUFA derivative, in combating AD.

#### 3.3.1. Docosahexaenoic Acid and Aβ

ω-3 PUFAs, particularly DHA and its derivatives, have shown multiple mechanisms capable of inhibiting the formation and neurotoxic effects caused by Aβ. DHA can hinder the formation of neurofibrillary tangles through the alteration in the nucleation and elongation phases, as well as the increased genetic expression of insulin-degrading enzyme (IDE), which mediates Aβ degradation [55,56]. Furthermore, dietary supplementation with DHA can reduce Aβ density in the hippocampus and toxic prefibrillar Aβ oligomers; additionally, it can stabilise soluble fibrillar Aβ oligomers in transgenic models performed in APP/PS1 rats with AD [57]. Additionally, DHA modulates Aβ 16-21 peptide aggregation by redirecting Leu-17, Phe-19, and Phe-20 residues, which eventually form stable and non-structured complexes, according to an extensive molecular dynamics simulation study [58].

In this sense, DHA inhibits the in vitro fibrillation of Aβ 25-35 and neutralises the neurotoxicity related to fibrillar formation [59]. On the other hand, DHA molecules exert anti-amyloidogenic effects on Aβ-42, the most abundant protein linked to amyloid plaque formation in AD [60]. Among the mechanisms involved, the ability of DHA to modulate the amyloidogenic and non-amyloidogenic processing of the APP is highlighted; it could be attributed to the induction of anti-amyloidogenic chaperones such as SorLA and transthyretin based on in vivo models performed in mice [61,62].

**Figure 2:**
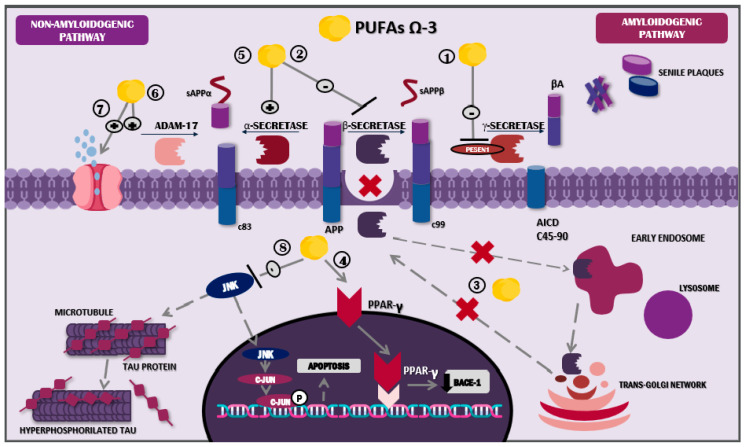
Mechanism of action of docosahexaenoic acid in the nervous system. Abbreviations: sAPPβ: soluble peptide APPβ, sAPPα: soluble peptide APPα; AICD: amyloid precursor protein intracellular domain; Aβ: amyloid beta; JNK: Jun N-terminal kinase; ω-3 PUFAs: omega-3 polyunsaturated fatty acids.

One of the main actions of DHA in amyloidogenic processing is the modulation of enzymes implicated in its metabolism. DHA can induce the suppression of PESEN1 and thus γ-secretase [63], as well as the inhibition of β-secretase. On the contrary, DHA stabilises the activity of α-secretase, enhancing a non-amyloidogenic process [64]. Furthermore, DHA modifies BACE1 internalisation in the endosomes, reducing β-secretase activity and subsequently inhibiting amyloidosis, typical of AD [64,65]. Similarly, DHA binds to PPARγ, activating these receptors and leading to reduced transcription and BACE1 expression [66,67] (Figure 2).

DHA also diminishes PESEN1 levels in lipid droplets, directly influencing amyloidogenic processes [64]. Additionally, it plays a role in cholesterol microdomains located on the membrane of the droplet, altering its organisation and structure; in other words, it boosts the change from droplet to non-droplet [68]. This process results in a loss of Aβ affinity for lipid droplets, leading to decreased accumulation of this element on neuronal plasma membranes [69].

Furthermore, DHA’s role in non-amyloidogenic processing involves increased levels of ADAM metallopeptidase domain 17 (ADAM17) and, consequently, of soluble amyloid precursor protein-alpha (sAPPα), a molecule associated with neuroprotective effects and β-secretase regulation [64,70]. In a similar manner, the potential activity of the lymphatic system through aquaporin-4 has been described; these molecules showed the ability to promote interstitial Aβ removal, and this mechanism was evidenced in an in vivo study using murine models treated with fish oil, one of the main dietary ω-3 PUFA sources [71] (Figure 2). Likewise, DHA has proven to possess a genetic role, since in vitro studies indicate that its consumption has inhibitory effects on the pro-amyloidogenic activity of the APOE ε4 allele [72].

Among the DHA actions against Aβ, microglial phagocytosis plays an important role, stimulating the activity of the anti-inflammatory phenotype M2 and subsequent Aβ-42 phagocytosis [44]. SPMs derived from DHA exhibit similar mechanisms: MaR1 and RvD1 stimulate Aβ phagocytosis via macrophages and microglial cells that bind to specific receptors for each molecule. Said actions provoke increased intracellular calcium levels and the activation of signalling pathways such as PPAR, extracellular signal-regulated kinase (ERK), protein kinase A (PKA), and phosphoinositide 3-kinase (PI3K) [72,73,74].

Nevertheless, the key SPM against Aβ is NPD1. The actions of NPD1 are very similar to DHA regarding amyloidogenic and non-amyloidogenic processing pathways, including PESEN1 reduction, the modulation of secretases, the augmentation in SorLA/LR11 expression, the down-regulation of BACE1, and the up-regulation of disintegrin and metalloproteinase 10 (ADAM10) activity, in addition to increased PPARγ-mediated activities. Each one of these mechanisms attributed to NPD1 is likely to result in Aβ and sAPPβ decrement, along with increased sAPPα levels [72,73,74], hence stimulating NPD1 synthesis and release, with subsequent positive feedback and long-term neurotrophic benefits [73].

Furthermore, Aβ deposits are linked to neuronal impairment and apoptosis. PPARγ activation by NPD1 can generate anti-apoptotic activities via the up-regulation of anti-apoptotic genes that codify the proteins Bcl-2, Bcl-xl, and Bfl-1 [75]. However, NPD1 is not the only molecule to exhibit anti-apoptotic properties; MaR1 has been implicated in the downregulation of pro-apoptotic proteins such as p38, caspase-3, and mTOR, thereby contributing to the inhibition of neuronal apoptosis [76].

#### 3.3.2. Docosahexaenoic Acid and Tau Protein

Since neurofibrillary tangles are a feature of AD, strategies that inhibit the aggregation of tau proteins or tau kinases to avoid or diminish disease development have been suggested [77]. It has been proposed that ω-3 PUFAs, and DHA specifically, could contribute as potential therapeutic agents with the capacity to inhibit the main kinases involved in tau protein phosphorylation, such as glycogen synthase kinase-3 beta (GSK-3 beta) and Jun N-terminal kinase (JNK) [78].

Experimental in vivo studies carried out in murine models with AD have shown increased JNK activity in the plaques of the neurites that contain phosphorylated tau, as well as a subsequent decrement after DHA supplementation. JNK is a kinase, part of the MAPK family, that can phosphorylate two sites of tau proteins related to AD: ser202/Thr205 and Ser422. The phosphorylation of these elements has been observed in early stages of the disease and is thought to precede neurofibrillary tangle formation [79]. DHA through the Mfsd2a transporter can directly inhibit JNK, leading to a decrease in p-c-Jun levels and thus lower tau hyperphosphorylation [80].

In contrast, DHA acts favourably on the PI3-K/Akt pathway, inhibiting GSK-3 beta and subsequently tau phosphorylation [78]. The GSK-3 enzyme can phosphorylate tau protein in 42 sites, and its activity is regulated by phosphorylation in two specific sites, serine 9 and tyrosine 216, allowing for its inactivation and activation, respectively. In AD, this regulation is altered, promoting the hyperactivity of the enzyme and leading to tau hyperphosphorylation [81].

### 3.4. Clinical and Epidemiological Evidence of Omega-3 Fatty Acids in Alzheimer’s Disease

Alzheimer’s disease is one of the main causes of dementia, and owing to the impact that it exerts over the patient’s life and its family environment, multiple strategies, such as dietary intervention, pharmacological treatments, gene editing, and monoclonal antibody therapies, have been considered to approach this pathology [82,83,84,85,86]. The nutritional role has gained particular interest. It was observed, in various epidemiological studies regarding Mediterranean diets rich in ω-3 PUFAs, a link between the mentioned dietary lifestyle and a lower prevalence of dementias and specifically AD [87,88,89,90,91,92,93]. This section will focus on discussing clinical and epidemiological findings related to ω-3 PUFAs as potential therapeutic agents in AD.

Under this premise, a cross-sectional study performed in 894 adults above ≥50 years old; it explored the association between diet, lifestyle and cognitive function, respectively, and showed that diets with abundant nuts and vegetables rich in ω-3 PUFAs had beneficial effects on cognition [94]. Moreover, a meta-analysis assessed the dose–response effect of ω-3 PUFAs in adults over 40 years old with normal cognitive functions or mild cognitive impairment. A significant improvement was evidenced 12 months after the intervention with doses over 500 mg. These results were attributed to the effects of ω-3 PUFAs on synaptic plasticity and neurogenesis in cerebral regions exposed to oxidative stress [95].

Likewise, another cross-sectional clinical trial linked greater cortical thickness (reduced in AD pre-symptomatic individuals) and a lower dementia risk to diets such as the Mediterranean diet and ω-3 PUFA-rich diets [96]. Similar results were observed in the Framingham Heart Study [97] and the Atherosclerosis Risk in Communities study [98]. Furthermore, Baierle et al. [99] demonstrated that individuals with diminished levels of ω-3 PUFAs, particularly DHA, exhibited reduced cognition compared with controls, thereby reinforcing the epidemiological evidence previously mentioned. However, this study is limited by a small population sample.

On the other hand, to examine the relationship between diet and AD brain biomarkers, Mosconi et al. [100] showed an association between greater ω-3 PUFA administration and less Aβ peptide development via brain imaging analysis. In addition, the authors have found that low ω-3 PUFA levels in serum were linked to increments in cerebral amyloidosis, while higher levels were correlated with the preservation of brain volume in AD-implicated regions [101]. In this sense, significant correlations were observed between the ω-3 PUFA index and entorhinal cortical volume, a structure tightly associated with the hippocampus and involved in memory and learning, as well as total white matter volume and decreased oxidative stress and inflammation [102].

Consistently, Wei et al. [103] carried out a meta-analysis that pointed out a potential modulating effect of APOE ε4, the most influential genetic factor in AD, through ω-3 PUFA supplementation. Similarly, the efficacy of these supplements showed a threshold concentration of 1.0 g/dL, since cognitive impairment risk declined once the supplementation exceeded this concentration. On the other hand, Sala-Vila et al. evidenced statistically significant associations concerning ω-3 PUFA levels and lower risk of AD and dementia in males and patients ≥60 years of age, in comparison with females between 50 and 59 years of age, suggesting that further investigation is needed due to the lack of clinical trials that effectively compare gender and age [104].

Nevertheless, despite the inverse relationship observed between ω-3 PUFA intake and AD risk in observational studies, the results of clinical trials are controversial. This phenomenon could be explained by factors such as inconsistencies in sample size, methodology, and time or by differences among the interventions [105]. Despite multiple studies showing the beneficial effects of supplementation, other clinical trials do not report any favourable results.

The Multi-domain Alzheimer Preventive Trial (MAPT) is a multicentric, randomised, placebo-controlled study and one of the biggest randomised clinical trials (RCTs) in this area. The MAPT aimed to assess the effects of ω-3 PUFA supplementation (two capsules a day providing a total daily dose of 800 mg of docosahexaenoic acid and 225 mg of eicosapentaenoic acid) on the cognitive function of adults over 70 years old without dementia. Beneficial effects were not demonstrated compared with the placebo group; however, a subgroup population analysis suggested that ω-3 PUFA supplementation, along with multi-domain intervention, could aid in slowing cognitive impairment in patients with a greater dementia risk [106].

It is important to note that other RCTs and secondary analyses of the MAPT have reported similar results, including less cognitive decline, improved orientation, and delayed recall [107,108]. Moreover, a recent clinical trial demonstrated the protective effect of ω-3 PUFA supplementation in individuals with cognitive deficits and dementia. These participants had family-style meals containing an additional 1720 mg of docosahexaenoic acid per day for 12 months [109]. Comparatively, a seven-trial RCT meta-analysis showed that compared with the placebo, ω-3 PUFA supplementation diminishes cognitive deficit significantly in elderly individuals (IC 95%: 0.04–1.67; *p* = 0.04) [110]. Additionally, various meta-analyses have found similar associations between ω-3 PUFAs and lower cognitive deficits. Overall, an 8 g/d increment in dietary PUFAs was significantly associated with lower risks of dementia [111,112] and AD risk [113] (Table 1).

On the contrary, an RCT secondary to the MAPT did not find significant effects on the cognitive functions of elderly patients subjected to a multi-domain intervention along with ω-3 PUFA supplementation over 36 months [117]. Similar results were found by Phillips et al. [114], where an increase in EPA/DHA concentrations in the serum of subjects did not show benefits on cognition; however, these results can be debated due to the small sample size and short follow-up period. Simultaneously, a meta-analysis of three RCTs pointed out that no beneficial effects on cognition were observed after consuming ω-3 PUFA-rich diets or supplementation in patients with moderate or mild AD [114].

Despite being considered a potential therapeutic strategy against AD due to its molecular properties, clinical evidence of ω-3 PUFA supplementation is mixed and inconclusive. One significant problem is the heterogeneity among these studies. The regimen protocols have not been standardised yet, and they differ in their supplementation composition. Furthermore, some studies assess ω-3 PUFA supplementation based on plasma levels, while others only select participants with a high intake of dietary ω-3 PUFA foods.

Currently, the focus of systematic reviews based on RCT presents several limitations. These studies aim to assess the efficacy of clinical interventions, but lack reproducibility as a result of their strict inclusion criteria, non-randomised nature, and short-term follow-up periods [118]. Likewise, other limitations, such as the use of unequal supplementations, dose, time, population, and diagnostic tools, may explain the incongruence of these results.

## 4. Conclusions

Alzheimer’s disease is characterised by the formation of amyloid plaques and neurofibrillary tangles that lead to a chronic pro-inflammatory state, neuronal apoptosis, and oxidative stress. To combat this pathology, ω-3 PUFAs might be considered potential therapeutic agents due to the extensive neuroprotective functions they exert. These molecules exhibit antioxidant and anti-inflammatory properties through microglial modulation and SPM production. In addition, ω-3 PUFAs play a key role in synaptogenesis and neurogenesis. Moreover, these fatty acids induce protective effects against AD by counteracting Aβ synthesis and its neurotoxic effects and influence APP processing in order to promote anti-amyloidogenic activities. Lastly, ω-3 PUFAs seem to increase Aβ peptide phagocytosis in microglial cells and hamper tau protein hyperphosphorylation via GSK-3β and JNK inhibition.

So far, current clinical and epidemiological evidence has demonstrated a positive relationship between ω-3 PUFA supplementation and lower risk of cognitive impairment as well as AD development; nevertheless, some results have been inconclusive. Also, the regimen protocols have not been solidly established, but studies demonstrating promising results are based on higher-dosage protocols (e.g., 1000–2000 mg of EPA and DHA combined daily) and long-term supplementation. Thus, it is necessary to perform more randomised clinical trials, with similar supplementation types, doses, and study durations, taking into account that DHA renovation in the brain takes approximately 2 years. Finally, the study subjects should be selected and screening tools adapted according to the specific population.

Even though ω-3 PUFA supplementation shows promise, particularly in the early stages of AD, there are several key limitations to consider in this therapeutic approach. The benefits appear to be stage-dependent, with greater effects observed in individuals in early AD or mild cognitive impairment. Genetic factors, such as the presence of the APOE ε4 allele, may influence individual responses to treatment. Furthermore, there is no standardised dosing protocol, making comparisons across studies difficult. Additionally, therapeutic effects take months to appear, which may be costly and difficult for some patients to maintain over the long term.

It is important to emphasise that although our study focuses on ω-3 PUFAs as a novel therapeutic approach for AD, there are also emerging pharmacological therapies such as anti-amyloid monoclonal antibodies that can slow clinical decline by promoting amyloid clearance. Among these, aducanumab, lecanemab, and donanemab have received US Food and Drug Administration approval. These agents are administered intravenously and exert their effects by activating microglia and inducing them to engulf and consume amyloids [119].

## Figures and Tables

**Table 1:** Efficacy of ω-3 PUFA supplementation in individuals with cognitive impairment or AD.

Authors	Methodology	Results
Burckhardt M. et al. [114]	Meta-analysis of three randomised trials, comprising a total of 632 patients with mild to moderate AD, aiming to determine potential side effects of ω-3 PUFA supplementation.	No significant effects on cognition associated with n3 PUFA use was observed via MMSE assessment (DM 0.18; IC 95%: 1.05–1.41; 202 participants; two clinical trials; I2 = 0%) and ADAS-cog (DME −0.02; IC 95%: 0.19–0.15; 566 participants; 3 clinical trials; I2 = 0%). Daily life activities did not show modifications (DME −0.02; IC 95%: −0.19–0.16; 544 participants; two clinical trials; I2 = 23%).
Wu S. et al. [113]	A meta-analysis of 6 prospective clinical trials assessing the link between n3 PUFA supplementation or fish consumption and AD incidence.	The results did not show statistical significance between n3 PUFA intake and a lower AD risk. However, after evaluating the dose–response effect of 100 g of fish per week, the risk of AD diminished (RR = 0.89, IC 95%: 0.79–0.99), and neuroprotective effects were observed.
Zhu et al. [112]	Meta-analysis of 14 prospective clinical trials that evaluated the relationship between n3 PUFAs and AD, dementia, and MCI risk.	No significant correlations between n3 PUFA consumption and lower AD risk (RR = 0.91, 95% CI = 0.74–1.11) nor dementia (RR = 1.13, 95% CI = 0.64–2.01) were observed. Nevertheless, PUFAs intake was linked to significantly lower risk of MCI (RR = 0.86, 95% CI = 0.75–0.98).
Zhang Y. et al. [110]	Meta-analysis of 21 clinical trials that investigated the association of fish intake and n3 PUFAs with MCI risk.	Increments in fish intake (1 serving per week) were significantly linked to lower dementia (RR: 0.95; 95% CI: 0.90, 0.99) and AD (RR: 0.93; 95% CI: 0.90, 0.95) risk.
Andrieu S. et al. [106]	Multicentric, randomised, placebo-controlled study of 3 years of duration, performed in patients above 70 years old with cognitive impairment, without dementia. A supplementation above the recommended dose was used, without exceeding the 2 g per day maximum intake. The assessment was conducted through a composite Z rating that combined four cognitive spheres. In addition to assessing every component, we also conducted additional cognitive tests.	No statistical significant differences in cognitive impairment between the groups were observed: −0.05388 (−0.6800 to 0.5723; (−0.6800 to 0.5723; standard error, SE = 0.3192; *p* = 0.8660)) for the ω3 AGPI group, −0.3936 (−1.0217 to 0.2345; SE = 0.3180; *p* = 0.2192), for the multi-domain plus placebo intervention group, and −0.6017 (−1.2255 to 0.02222; SE = 0.2092; *p*= 0.3202) for the placebo group.
Hooper C. et al. [108]	MAPT secondary analysis. Two groups were selected: the placebo group and a subgroup of the n3 PUFA group, composed of individuals with a lower n3 PUFA index. The goal was to determine if the supplementation would benefit patients at risk of dementia.	The group with low n3 PUFA supplementation demonstrated improvements in the cognitive COWAT test compared with the control group (*p* = 0.009; the difference between the median of both groups was 2.3; IC 95%: 0.6–4.0).
Hashimoto et al. [109]	Randomised, double-blind, controlled clinical trial of 1 year of duration, performed in 75 adults aged 88.5 ± 0.6 years, assessed through HDS-R7 and MMSE scales, as well as the Japanese version of the Apathy Scale and the Zung Scale.	The results obtained demonstrated a significant improvement in the supplementation group compared with the placebo group in one of the subitems of the MMSE (F = 4.50, ε = 0.97, *p* = 0.01). Furthermore, mild although statistically significant modifications were observed in the apathy test (*p* = 0.04). These changes were attributed to dose, as modifications were less pronounced in individuals with low n3 PUFA supplementation.
Calderon Martinez et al. [115]	Meta-analysis of 14 studies on the effects of n3 PUFA and Souvenaid^®^ (medical nutritional drink with DHA, EPA, and other nutrients) supplementation in AD.	A total of 58% of the publications showed a favourable outcome with supplementation, with evidence of a decrease in cognitive impairment according to the CDR scale (MDS = −0.4127, 95% CI: [−0.5926, −0.2327]), with no differences between the type of intervention and the type of cognitive impairment (MDS = −0.4127, 95% CI: [−0.5926, −0.2327]).
Shinto et al. [116]	A 3-year, randomised, quadruple-blind, placebo-controlled clinical trial in 102 patients of >75 years of age without dementia or with mild cognitive impairment with WML greater than 5 cm and plasma omega-3 levels less than 5.5 percent by weight of total omega-3 to determine whether n3 PUFA supplementation with 1.65 g of ω-3 PUFAs (975 mg of EPA and 650 mg of DHA) reduces WML accumulation in patients with WML and suboptimal n3 PUFA status.	The omega-3 supplemented group had a smaller annual reduction in WML than the placebo group, but this difference was not statistically significant (1.19 cm^3^ [95% CI, 0.64–1.74 cm^3^] vs. 1.34 cm^3^ [95% CI, 0.80–1.88 cm^3^]; *p* = 0.30). However, among the APOE ε4 patients, the annual decrease in the marker of white matter integrity impairment was significantly lower in the ω-3-treated group than in the placebo group (−0.0016 mm^2^/s [95% CI, −0.0032 to 0.0020 mm^2^/s] vs. −0.0047 mm^2^/s [95% CI, −0.0067 to −0.0025 mm^2^/s]; *p* = 0.04).

Abbreviations: n3 PUFA: polyunsaturated fatty acid; MMSE: mini-mental state examination; CI: confidence interval; RR: relative risk; ADAS-COG: Alzheimer’s disease assessment scale–cognitive; AD: Alzheimer’s disease; MCI: mild cognitive impairment; COWAT: controlled oral word association test; MAPT: Multi-domain Alzheimer Preventive Trial; WML: white matter lesion.

## Data Availability

No new data were created or analysed in this study. Data sharing is not applicable to this article.

## Abbreviations

The following abbreviations are used in this manuscript:

ACH	Amyloid cascade hypothesis
APP	Amyloid precursor protein
ALA	Alpha-linolenic acid
Aβ	Amyloid-β
COX	Cyclooxygenase
DHA	Docosahexaenoic acid
DHEA	Docosahexaenoyl ethanolamide
AD	Alzheimer’s disease
EPA	Eicosapentaenoic acid
ERK	Extracellular signal-regulated kinase
GPCR	G protein-coupled receptors
GPCR40	G protein-coupled receptor 40
GSK-3 beta	Glycogen synthase kinase-3 beta
HO-1	Heme oxygenase 1
IDE	Insulin-degrading enzyme
IGF-1	Insulin growth factor
IκB	Inhibitory subunit of factor nuclear kappa B
IL-1β	Interleukin-1β
IL-6	Interleukin-6
JNK	c-Jun N-terminal kinases
CSF	Cerebrospinal fluid
LOX	Lipoxygenase
MAPK	Mitogen-activated protein kinases
Mar	Maresin
NGF	Nerve growth factor
NPD1	Neuroprotectin 1
NO	Nitric oxide
NF-κB	Nuclear factor kappa beta
PESEN1	Presenilin 1
PESEN2	Presenilin 2
PI3K	Phosphoinositide 3-kinase
PUFA	Polyunsaturated fatty acid
PKA	Protein kinase A
PKC	Protein kinase C
PPAR-γ	Peroxisome proliferator-activated receptor γ
PMN cells	Polymorphonuclear cells
ROS	Reactive oxygen species
RvEs	E series resolvins
RvDs	D series resolvins
sAPPα	Soluble amyloid precursor protein-alpha
SPM	Specialised pro-resolving lipid mediator
CNS	Central Nervous System
TLR	Toll-like receptors
TNF-α	Tumour necrosis factor alpha
VEGF	Vascular endothelial growth factor
